# The Kindergarten

**Published:** 1886-10

**Authors:** Jennie Merrill

**Affiliations:** The Normal College, New York City


### *THE KINDERGARTEN.*

Judging by external appearances, many say, "The kindergarten is to amuse children." They do not realize that it is a wonderfully rounded and complete method of education; that it sends its roots back deep into the home; and that Froebel had in mind "The Education of *Man*," not of the *child* merely.

The present danger seems to be a tendency to confound primary objective and busy works with the kindergarten. I approve of using some of the kindergarten material in teaching numbers, form and other lessons in the primary school. The busy work drawn so largely from kindergarten occupation is excellent. I only object when this work is supposed to take the place of the kindergarten itself. This it does not do. If I were teaching a primary class, I should be lost without my knowledge of the kindergarten. I should draw from it constantly for illustration, but I should not for a moment think I was teaching a kindergarten.

If Froebel had lived longer or begun his work earlier he would have in all probability carried forward certain of the occupations for higher work succeeding the kindergarten. This his disciples are now endeavoring to do. At present the work accomplished in the kindergarten is not generally used to the best advantage in the primary school.

Sometimes teachers are found who enter complaints against the little ones who come from the kindergarten, as "They want to be amused," "They are not able to work alone," "They are not well disciplined." If there are any such teachers here I want to ask them to withhold judgement. There are kindergartens and kindergartens. Such complaints are not found when children have been trained in good kindergartens. In such children are often left to work unaided. Their attention is not concentrated upon "making pretty things to take home." The gifts are preferred to the occupations as of greater educational value.

Too much is sometimes expected from the kindergarten. It cannot transform a child into a genius. It only encourages natural growth.—*Jennie Merrill, of the Normal College, New York City.*

